# On the Treatment of Hernia

**Published:** 1825-01

**Authors:** J. C. Yeatman

**Affiliations:** Surgeon to H. R. H. the Duke of Gloucester, Member of the Royal College of Surgeons, &c.


**Art. V.—*On the Treatment of Hernia.* By J. C. Yeatman, Esq. Surgeon to H. R. H. the Duke of Gloucester, Member of the Royal College of Surgeons, &c.**

In army and private practice, twenty-four cases of strangulated hernia have been treated by me with success; twenty-one of which were reduced by using the following means, and three were operated upon at an early period of the strangulation.—Fifteen of the above were cured by venesection, the application of cold, and the taxis; and six by these means, added to the internal exhibition of five grains of opium. The intestine, in all the twenty-four cases, had been strangulated from one to five hours. Each patient was immediately bled *ad deliquium*; then placed on the floor of the room, on a blanket, with pillows to raise the pelvis, the thighs being bent, and the taxis managed in the usual way. Pails of cold water, with ice, or muriate of soda and ammonia, dissolved in the water, were placed by the side of the patient, and poured on the hernial tumor, at the distance of two feet, from the spout of a large tea-pot or small tea-kettle, for a space of time varying from an hour to an hour and a half; occasionally employing the taxis for a few minutes together. When this mode of treatment failed of success, five grains of opium were given, in each case, the patient put to bed, and the taxis had recourse to during a profound sleep.

The above number includes all the cases of strangulated hernia which have fallen under my care, or for which I have been consulted; and the above are the only means which have been employed or recommended by me.

It is well known that cold, by condensing the rarefied air in the intestine, diminishes the volume; and that a large dose of opium produces muscular relaxation, particularly if the patient be bled largely prior to that drug being swallowed. I pass on, therefore, to observe, that, to use the lancet, cold, and opium, with far more success than common, they must, at least, be employed in the above manner, and to the above extent.

Concerning the operation for hernia, it may here be said, that it is most disgraceful to the surgeon to find the contents of the hernial sac in a state almost approaching to gangrene, when he might have operated at an *early* period of the strangulation: but, alas! all the remedies which have ever been recommended in such cases, are put in requisition, and many hours, and even days, are too frequently suffered to elapse in imbecile and reckless attempts to return the inflamed, enlarged, and strictured parts into the abdominal cavity.

It is now seventeen years since I had occasion to observe, before the Westminster Medical Society, that the surgeons of the Bristol Infirmary, (my uncle having been one of them, and myself a pupil at that excellent hospital,) usually employed, with the taxis, only three or four of the more powerful remedies to reduce the hernial tumor,—as venesection, cold, and tobacco; and that, if they did not succeed in the course of three or four hours, they proceeded to operate: and, ascribing the cause of their great success to the circumstance of operating at an early period of the strangulation, I well recollect that that eminent surgeon, Mr. Brodie, coincided with me in opinion, and both ably and warmly supported it.

***Frome; November*** **11*th*, 1824.**

**[We regret that our limits oblige us to postpone till next month the other Original Communications announced in our last Number.—EDITORS.]**

